# MeRy-B: a web knowledgebase for the storage, visualization, analysis and annotation of plant NMR metabolomic profiles

**DOI:** 10.1186/1471-2229-11-104

**Published:** 2011-06-13

**Authors:** Hélène Ferry-Dumazet, Laurent Gil, Catherine Deborde, Annick Moing, Stéphane Bernillon, Dominique Rolin, Macha Nikolski, Antoine de Daruvar, Daniel Jacob

**Affiliations:** 1 Université de Bordeaux, Centre de Bioinformatique de Bordeaux, Génomique Fonctionnelle Bordeaux, F-33076 Bordeaux, France; 2 INRA, UMR 1332 Biologie du Fruit et Pathologie, Centre INRA de Bordeaux, F-33140 Villenave d'Ornon, France; 3 Plateforme Métabolome-Fluxome Bordeaux, Génomique Fonctionnelle Bordeaux, IBVM, Centre INRA de Bordeaux, BP 81, F-33140 Villenave d'Ornon, France; 4 Université de Bordeaux, UMR 1332 Biologie du Fruit et Pathologie, Centre INRA de Bordeaux, F-33140 Villenave d'Ornon, France; 5 Université de Bordeaux, Laboratoire Bordelais de Recherche en Informatique, UMR 500, F-33405 Talence, France

## Abstract

**Background:**

Improvements in the techniques for metabolomics analyses and growing interest in metabolomic approaches are resulting in the generation of increasing numbers of metabolomic profiles. Platforms are required for profile management, as a function of experimental design, and for metabolite identification, to facilitate the mining of the corresponding data. Various databases have been created, including organism-specific knowledgebases and analytical technique-specific spectral databases. However, there is currently no platform meeting the requirements for both profile management and metabolite identification for nuclear magnetic resonance (NMR) experiments.

**Description:**

MeRy-B, the first platform for plant ^1^H-NMR metabolomic profiles, is designed (*i*) to provide a knowledgebase of curated plant profiles and metabolites obtained by NMR, together with the corresponding experimental and analytical metadata, (*ii*) for queries and visualization of the data, (*iii*) to discriminate between profiles with spectrum visualization tools and statistical analysis, (*iv*) to facilitate compound identification. It contains lists of plant metabolites and unknown compounds, with information about experimental conditions, the factors studied and metabolite concentrations for several plant species, compiled from more than one thousand annotated NMR profiles for various organs or tissues.

**Conclusion:**

MeRy-B manages all the data generated by NMR-based plant metabolomics experiments, from description of the biological source to identification of the metabolites and determinations of their concentrations. It is the first database allowing the display and overlay of NMR metabolomic profiles selected through queries on data or metadata. MeRy-B is available from http://www.cbib.u-bordeaux2.fr/MERYB/index.php.

## Background

The set of low-molecular weight (usually < 1500 Da) molecules of an organism, organ or tissue is referred to as the metabolome [1], and the comprehensive qualitative and quantitative analysis of this set of molecules is called metabolomics [2]. Metabolome analyses aim to provide a holistic view of biochemical status at various levels of complexity, from the whole organism, organ or tissue, to the cell, at a given time. Metabolomics is increasingly widely used by plant biologists [3-6] studying the effects of genotype and biotic or abiotic environments [7-9] or the biochemical modifications associated with developmental changes [10,11]. It is also widely used by food scientists, for descriptions of changes in the organoleptic properties and nutritional quality of food [12] and evaluations of food authenticity [13]. It is also used in substantial equivalence studies for genetically modified organisms [14]. Metabolomics has also increasingly entered into routine use in plant functional genomics, in which correlations between such biochemical information and genetic and molecular data are improving our insight into the functions of unknown genes [15-17]. Finally, it is emerging as a tool for the screening of genetic resources and plant breeding [18,19].

The chemical diversity and complexity of the plant metabolome constitutes a real challenge, even for a given species, because the diversity of metabolites and their concentration ranges remains huge. It is therefore impossible to profile all metabolite families (the list of these families includes amino acids, organic acids, carbohydrates, lipids and diverse secondary metabolites, such as phenylpropanoids, isoprenoids, terpenoids and alkaloids) simultaneously through a single extraction and with only one analytical technique. Most metabolomics projects therefore use several analytical strategies in parallel [17,20]. Several techniques of choice have emerged, including gas chromatography or liquid chromatography coupled with mass spectrometry (GC-MS or LC-MS) and proton nuclear magnetic resonance spectrometry (^1^H-NMR) [21,22].

^1^H-NMR and GC-MS have been applied to polar extracts for the study of primary metabolites. ^1^H-NMR technology has been widely used as a high-throughput technique for non targeted fingerprinting with little or no sample preparation [23,24]. It has also been applied to targeted profiling and the absolute quantification of major metabolites [25], despite its relatively low sensitivity, taking advantage of its large dynamic range [22]. GC-MS is much more sensitive than ^1^H-NMR and is ideal for the detection of volatile metabolites, but high-boiling point metabolites require two-step derivatization [26].

The relative quantification of a hundred hydrophilic metabolites can be achieved, but comparisons of sets of GC-MS metabolomics profiles obtained in different laboratories remain difficult. For the study of secondary metabolites, LC-MS analysis is generally the method of choice. Extracts are injected directly, without derivatization. LC-MS is generally used for metabolomic profiling [27] with relative quantification. The use of shared databases is hindered by cross-compatibility problems between spectra acquired with different LC-MS instruments [28], even with two instruments of the same model from the same manufacturer. High-resolution MS techniques, such as FT-ICR-MS, are also used without LC separation and are very promising for use in plant metabolomics [29]. However, a complementary technique, such as NMR, is often required for further characterization of specific metabolome changes in terms of structure [30]. A major advantage of ^1^H-NMR is that the profiles obtained are often comparable, even between different instruments or different field magnitudes [31,32], provided that some parameters, such as extract pH, are fixed at a constant value.

Metabolomics facilities, including those using ^1^H-NMR, generate large amounts of raw, processed and analyzed data, which must be well managed if they are to generate useful knowledge. Various web-based software platforms are available for managing and making use of metabolomics data. These software platforms include metabolite spectral databases, such as the Golm Metabolome Database (GMD) and the Human Metabolome DataBase (HMDB). The GMD [26] provides public access to GC-MS data and peak lists for plant metabolites. The HMDB [33,34] is an example of an organism-specific database providing detailed information, including quantification and information about the spatial distribution of small metabolites in the human body. These metabolite-oriented platforms also provide simple query forms for searches by mass or compound names. Standard compound libraries, such as the Biological Magnetic Resonance data Bank (BMRB) [35] are also useful for metabolite identification by NMR. Databases of this type may be seen as knowledgebases rather than integrated tools for data management, analysis and metabolite identification. MeltDB [36] and SetupX [37], two web-based software platforms for the systematic storage, analysis and annotation of datasets from mass spectrometry (MS)-based metabolomics experiments, have recently been implemented. However, these platforms cannot handle NMR data. Another platform, PRIMe [38], provides standardized measurements of metabolites by multidimensional NMR spectroscopy, GC-MS, LC-MS and capillary electrophoresis coupled with MS (CE-MS). It also provides unique tools for metabolomics, transcriptomics and the integrated analysis of a range of other "-omics" data. The standardized spectrum search in PRIMe is a very useful tool, but it does not provide information about the biological context of compounds, unlike the KNApSAcK database linking metabolites identified by MS to species http://www.metabolome.jp/software/knapsack-database or Phenolexplorer [39], a bibliographic database http://www.phenol-explorer.eu dedicated to the polyphenol content of food. MetaboAnalyst [40] is an online tool for processing high-throughput metabolomic data from NMR and GC/LC-MS spectra. For NMR, it allows statistical analysis of compound concentration data obtained by quantitative metabolic profiling or of ^1^H NMR spectral signatures (after data reduction with bucketing) for urine samples for example. MetaboAnalyst does not handle NMR spectra but only processed data (peak list or buckets list) in tabular csv files. Each of these applications is useful, but none constitutes a complete tool for managing, analyzing and sharing plant NMR metabolomics data.

Given the types of metabolomics resources available (listed in [34]), and the key aspects of both the analysis and understanding of metabolomics data (identified as Visualization in [41]), there is currently a need for *i*) a spectral database combined with *ii*) a knowledgebase for plants, *iii*) an easy-to-use metabolomic spectral visualization tool and *iv*) a metabolomic data analysis tool. Taking these requirements into account, we have developed a plant metabolomics platform (with public or private access) for the storage, management, visualization, analysis, annotation and query of NMR fingerprints or quantitative profiles and quantified metabolite. This platform has been named MeRy-B, for Metabolomics Repository Bordeaux. MeRy-B facilitates profile discrimination through the visualization of spectral data by either modular spectrum overlay (*i.e*. driven by the choice of criteria or factors from the experimental design) or multivariate statistical analysis. It can also construct a knowledgebase of plant metabolites determined by NMR, including metabolite concentration data when available, with minimal information about experimental conditions in the context of scientific publications, and can be used for the re-analysis of combined experiments. Furthermore, MeRy-B provides tools for the identification of metabolites by comparisons of spectra for plant extracts with spectra available in the MeRy-B knowledgebase.

## Construction and Content

### Standards for metabolomics

Data storage and database building tools are required for the storage and analysis of present and future metabolomics data. MeRy-B therefore takes into account the recommendations of initiatives concerning the extent and types of metadata (information associated with the data or data about the data) to be stored for each metabolomics experiment: MiAMET [42,43], Standard Metabolic Reporting Structure (SMRS) [44], Metabolomics Standard Initiative (MSI) [45]. In terms of plant biological context, MeRy-B also includes a small number of parameters required to define the experimental study design [46].

### MeRy-B database design

**Figure 1:**
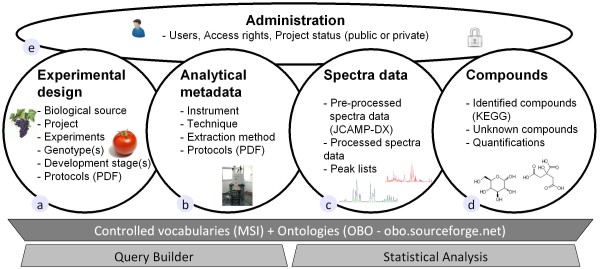
**MeRy-B architecture and workflow for the capture and management of metabolomic data**. MeRy-B has four components, following the steps of a metabolomic experiment: (a) description of Experimental Design, (b) Analytical Metadata, (c) Spectral Data, including preprocessed spectra data supplied by users and processed spectra obtained with custom-designed tools, (d) capture of Compounds with names based on the KEGG database and chemical annotation of chemical shift based on IUPAC rules where possible. Metadata description is supported by controlled vocabularies and ontologies. Unstructured "free" text is recorded as protocols in PDF format. The administration component (e) takes into account different rights of access for both projects and users. Project status defines the type of information to which users have access, as a function of their access rights for the project concerned.

The architecture of MeRy-B (Figure 1) is based on the ArMet model [43,47] and MIAMET/MSI requirements [42,48]. We improved the 'volume of information inserted by user'/'time spent to insert' ratio by deciding to store a minimum of information in the database. MeRy-B therefore contains fewer components than ArMet. The aim of this compromise was to ensure that only the most relevant metadata are stored. Controlled vocabularies are proposed, where possible, to standardize the information recorded and to reduce the time required to input information.

Additions to the database are made principally through web interfaces, with various forms. These data input forms are accessible to registered users. Other metadata are uploaded, stored in files and made available for consultation. For example, all protocols are collected in PDF format files, as such files are already available as part of the quality assurance approach operating in most laboratories: standard operating procedures (SOPs) are available and users therefore waste little time uploading these data into the MeRy-B database.

The database is structured according to the steps in a metabolomics experiment and therefore consists of four principal components: "Experimental design" (Figure 1a) "Analytical Metadata" (Figure 1b), "Spectra data" (Figure 1c) and "Compounds" (Figure 1d). There is also a fifth component: "Administration" (Figure 1e). Unlike MeltDB [36], MeRy-B is based on the description of an experiment according to the logic of the metabolomics approach (Figure 1). Thus, experimental context is the first subject tackled, and spectra are then allocated to this biological context.

### Experimental metadata

The Experimental Design component describes the biological source and protocols for plant growth, sample harvest, extract preparation and storage (Figure 1a). The experimental details are crucial for data interpretation and use in subsequent studies, so all metadata relating to experimental design are described in detail. For this purpose, descriptions are based, as far as possible, on controlled vocabularies and ontologies, such as NCBI Taxonomy http://www.ncbi.nlm.nih.gov/Taxonomy/, Plant Ontology Consortium http://www.plantontology.org/ and Environment Ontology http://environmentontology.org/. A *Project *is defined as an entity comprising a set of experiments carried out on one species by a laboratory, at a particular geographic site. Within a given *Project*, each *Experiment *is carried out within a particular set of environmental conditions, such as '*control*' or '*stress*'. A protocol file in PDF format is uploaded for each step in the experiment: *growth*, *harvest *and *storage *of the biological samples. Five types of biological factor potentially contributing to definition of the experimental design are defined: organ or tissue, genotype, genetic background, developmental stage and environmental conditions.

### Analytical metadata

MeRy-B also manages metadata concerning the analytical part of the experiments. The preparation of analytical samples (plant extracts or plant fluids, such as sap or exudate), parameters of analytical instruments and spectrum processing metadata are described in PDF protocols (Figure 1b). The PDF file for Extraction also contains information about the number of samples and the way they were coded, including the parameters of biological and technological replicates. The descriptions of extraction methods and analytical instruments are stored into the database on forms, allowing these metadata to be queried. Each item of analytical metadata is linked to an analytical technique (*i.e*. ^1^H-NMR).

MeRy-B can generate *Analytical Profiles *to assist the user with the input of repetitive analytical metadata. An Analytical Profile consists of an instrument description, an extraction method description and the various types of protocol: extraction, analytical and processing.

### Spectral data

The *Spectral data *component describes spectrum format and processed data (Figure 1c). MeRy-B supports the standard ascii exchange format for spectroscopic data: JCAMP-DX for ^1^H-NMR spectra. Spectra in proprietary formats (Bruker, Jeol, and Varian) must be converted into JCAMP-DX format (1r 1 spec: real processed data). Spectra may be uploaded as data that have already been preprocessed by commercial software (Fourier Transformation, manual phasing and baseline correction). Alternatively, MeRy-B provides custom-designed signal processing methods for 1r NMR data. These methods include noise suppression, baseline correction (signal denoising and baseline correction are obtained by discrete wavelet transform [49]), deconvolution (searching for peaks from the third order of signal derivative, building a modeled spectrum as a sum of Lorentzian shapes, followed by an optimization step based on the Levenberg-Marquardt algorithm [50]) and the automatic detection of chemical shift indicators (*i.e*. TSP or DSS). Each spectrum, whether modeled or not, is linked to an Experimental Design and an Analytical Profile.

### Compounds

The *Compounds *component provides information about the identification of a given compound and its quantification, when available (Figure 1d). Each spectrum can be linked to a compound list, with compound chemical shifts and quantifications, when available. The user may declare a compound as "known", with KEGG IDs and names (KEGG compound database http://www.genome.jp/kegg/compound/[51]), or as "unknown". In the MeRy-B database, an unknown compound is a compound with an unknown structure but a known 1D ^1^H-NMR signature (pattern of the NMR signal: singlet, doublet, triplet or multiplet, and their chemical shifts). A specific nomenclature is used to allocate identifiers to the unknown compounds, to link these unknown signatures in the various spectra of the database. For example, an interesting singlet peak was detected on a spectrum at 1.9 ppm. This unknown compound is thus named unkS1.90: with S for singlet and 1.90 for the chemical shift expressed in ppm in agreement with the recommendations of MSI [48]. A putative identification may be added as a comment. The user is free to add comments to all the compounds identified as known and unknown.

### Administration

The database also contains an *Administration *component (Figure 1e), to manage the accounts and access rights of users at project level. The "Admin user" has the right to create new entities, such as Instrument, Localization, and Controlled Vocabulary, such as genotype.

The user responsible for creating a project automatically becomes its "owner". The owner of a project can provide temporary or permanent access rights (insertion, deletion of data) to other users on his or her project. By default, a *project *is private. However, it may be made public (for consultation only) if access via the public user account is set up by the project's owner.

### Database implementation

MeRy-B is a PostgreSQL relational database accessible through a web interface developed in the PHP language. The web interface is rendered dynamic by the use of JavaScript and AJAX technologies. The application is maintained on a Linux server. A Java applet has been developed for ^1^H NMR spectrum visualization (the self-signed certificate is available on the"About MeRy-B" page). The backend statistical computing and visualization operations are carried out with functions from the R packages and Perl scripts. Data storage, treatment and querying have been developed with Perl, XML and web services technologies, such as SOAP.

## Utility and Discussion

MeRy-B fulfills two needs. First, each registered user, as a project owner, creates projects and deposits his or her own data and associated metadata into the application for storage, consultation, visualization and analysis. At this point, there is no curation team deciding whether or not an upload should be allowed. However, the administrator is alerted when a project is rendered public and he verifies this new inclusion of data. Second, all users are allowed to search the MeRy-B knowledgebase constructed from the information provided by all previous project owners (public data), for the re-analysis and comparison of data sets and to facilitate compound identification. The utility of MeRy-B for each of these cases is detailed below. A user manual illustrated with screenshots is available from the MeRy-B website for a more detailed description.

### How to upload and consult a metabolomics project on MeRy-B as project owner

Data uploading and consultation are illustrated here, as a use case, with the data and metadata of a published study on tomato [10]. Four main types of data were entered through the *Data capture *module in the MeRy-B database: (1) experimental design, (2) analytical metadata, (3) spectral data, and (4) compounds (lists and/or quantifications). Three main steps were used 1) creation of the users account and project, 2) population of the database with the user's data, and 3) analysis and visualization of the user's data. The aim of the tomato study was to characterize differences between the metabolic profiles of two interdependent tissues, seeds and flesh, from the same fruits, during fruit development, by means of a metabolomics approach. Before the creation of the MeRy-B project, it was necessary to define an informative title and to decide which factors should be taken into account for subsequent data visualization and analysis. Two factors, tissue (Seed vs Flesh) and developmental stage, were clearly identified and guided the coding of the biological samples and the organization of the data in the database. Two experiments were created: Tomato-Seed and Tomato-Flesh.

Once the user's account had been created by the MeRy-B administrator, an accession number was allocated: T06002 (T for tomato, 06 for year 2006 and 002 for the second project on tomato in 2006). The project was created by uploading the three protocols describing *Growth*, *Harvest *and *Storage *as pdf files through the *Protocols *menu: PG- Tomato - Metabolomics - 2006, PH- Tomato - Metabolomics - 2006 and PS-Tomato-UMR619-1. The 'Environmental Condition', 'Study Type' and 'Tissue/Organ' were selected from drop-down lists: Normal, Growth chamber study and Seed or Fruit. Several controlled vocabularies were also required, such as *Culture Localization*, *Genotype Lycopersicum esculentum *var 'Ailsa Craig'. These requests were sent to the MeRy-B administrator who created and added this new controlled vocabulary. The five *Developmental stages *were then created by the user for each experiment: from FF.01 fruit size 30% (8 days post anthesis or DPA) to FR.04 fruit ripening complete (45 DPA) and the genotype was chosen (Ailsa Craig). The *Analytical Metadata *component was then created and documented with a description of the NMR spectrometer (in *Instrument *Menu), NMR sample preparation (conditions of sample preparation by resuspension or reconstitution in solvent (in the *Methods *menu)), the protocols used for extraction/preparation of the samples (PE-Tomato - Metabolomics -2006), NMR acquisition (PA- Tomato - Metabolomics -2006) and NMR processing (PP- Tomato - Metabolomics -2006). The next step was the creation of *Analytical Profiles*. Sample coding was described in the extraction protocol: e.g. Sx.y.z indicates Seed sample at x days post anthesis, y indicates the pool or biological replicate number and z, the technological replicate. During the transformation of NMR spectra from Bruker format to JCAMP-DX format, the spectra were renamed with the above code. They were then imported into MeRy-B through the *Spectral Data *module.

**Figure 2:**
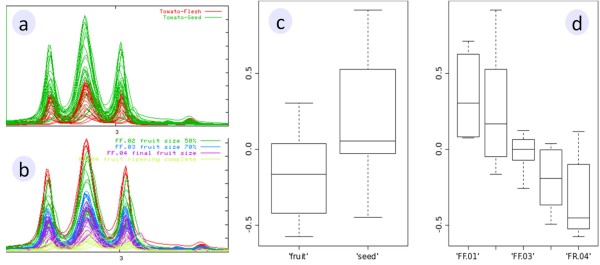
**Example of the MeRy-B NMR Spectra overlay and Statistical visualization tool**. Overlay of a portion of the NMR spectra colored according to the tissue (Flesh vs Seed, (a)) or developmental stage (b) criterion. (c) and (d) illustrate the ANOVA results of the spectral region centered on 3 ppm (bucket size 0.04 ppm) as a box and whisker plot representation. These box and whisker plot representations provide a graphical view of the multiple comparison results based on the tissue (c) or developmental stage (d) criterion.

During the third step, within the *Data consultation *menu, the overlay module was particularly useful for checking the quality of spectra and the View module for checking the consistency of biological replicates. In addition, as spectra are colored according to criteria chosen by the user, such as by experiment, developmental stage or sample code, visual inspection and identification of the spectral areas specific to a tissue (Figure 2a) or a stage of development (Figure 2b) was facilitated by this overlay module, which is much more powerful than the dual function based exclusively on sample code provided by the manufacturers of NMR software. For instance, with MeRy-B *Spectra overlay*, (Figure 2a and 2b) it was possible to identify developmental stage biomarkers (e.g. doublets at 7.66, 7.21, 7.13, 6.96 and 6.4 ppm, subsequently identified as chlorogenic acid; and a multiplet at 1.9 and two triplets at 2.3 and 3.01 ppm, subsequently identified as gamma-aminobutyric acid or GABA) or tissue biomarkers (e.g. doublets at 5.44 and 5.00 ppm, putatively identified as a planteose-like compound, a major oligosaccharide in tomato seed).

In addition to visual inspection, MeRy-B statistical tools were applied to regions of the spectral signature or buckets (data reduction using bucket size of 0.04 ppm, bucket intensity normalized to total intensity; and water signal region excluded from 4.97 to 4.7 ppm). These tools included standardization of bucket intensities followed by principal component analysis (PCA) or analysis of variance (ANOVA) (Figures 2c and 2d), for the identification of relevant spectral regions [52] and help in targeting of the metabolite identification process.

This MeRy-B output for the T06002 tomato project was consistent with the findings of the previous study [10], which highlighted the same developmental stage biomarkers by a different approach: PCA and comparison of the means of absolute quantifications for the identified metabolites with SAS version 8.01 software.

**Figure 3:**
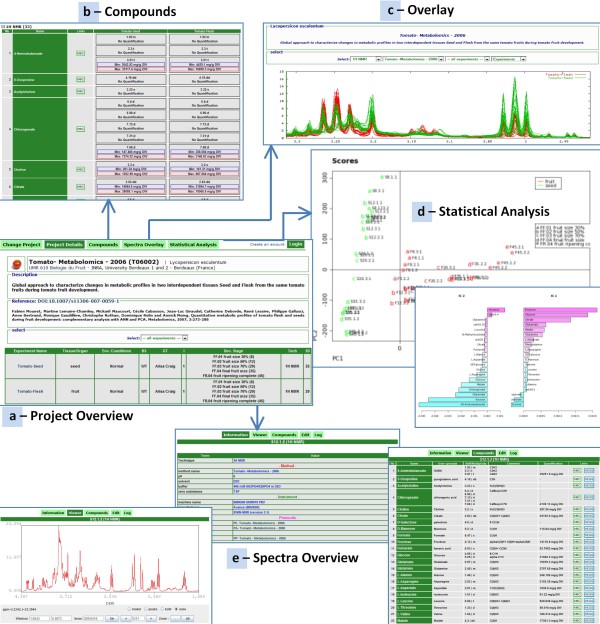
**Examples of Visualization and Statistical Analysis results for the tomato project T06002**. Screenshots from the various visualization and statistical tools. The user selected the tomato project T06002 (a), the composition overview of the samples (b), visualization of the NMR spectra according to tissue criteria (c), visualization of the statistical analysis results (d) and a zoom on one specific spectrum (e). MeRy-B provides statistical analysis facilities within each project. First, the experimental factors and individual samples (rows) and the spectral region variables (columns) for construction of the initial data matrix must be chosen. Second, a statistical analysis workflow must be selected from a list of proposals. Workflow typically begins with standardization of the data, followed by data reduction by analysis of variance (ANOVA) to select the meaningful variables (p-value threshold 0.05). An unsupervised method, such as principal component analysis (PCA), can then be used, if desired, to determine a set of variables from the inputs that can be used to classify the samples into factor groups. An ANOVA test can then be applied to each variable of the set, generating box and whisker plots making it possible to check the relevance of the discrimination. If variables are of the analytical type, it may be important to ensure that they are not affected by an analytical artifact (such as chemical shift). Such checks can be carried out with the Spectra overlay tool, which can be used to visualize all the spectra of an experiment, overlaid in a single graph.

In addition, known or unknown compounds identified on NMR spectra in [10] were documented in MeRy-B, by selecting the menu *Compound*, and then *Add compound*. The list of identified and/or quantified metabolites established was downloaded via '*Download the quantifiable compounds list*' and opened with spreadsheet software on a PC (e.g. MS Excel) for completion with the quantification data from each NMR spectrum. This file was then uploaded into MeRy-B. The quantitative data can be visualized for the entire T06002 project through the menu *Data consultation*, *Projects*, *Compounds *(Figure 3b) or for each spectrum, by selecting the spectrum and the *Compounds *menu (Figure 3e).

At this point, the project owner decided to share the data with the scientific community. In most cases, this occurs at the time of publication of the corresponding paper. Therefore, the reviewers will have had the opportunity to check the quality of the spectra and the metadata during the review process, as they will have been provided with special logins. The curation process is therefore partly carried out by the reviewers of the scientific journal. Nevertheless, when the project owner renders the data publicly available, the system alerts the administrator and allows him or her to curate the data and to validate the definitive inclusion of the data into MeRy-B.

### Consulting a metabolomics project on MeRy-B

Once a project has been imported and rendered public (*i.e*. after publication), the experimental data and related metadata can be consulted through the *Data consultation *module and its various interfaces, providing either a global view or a detailed view. The complete experimental design, by project, is available through the *Project Details *function, which provides an overview on a single web page (Figure 3a). From this web page, a global view of each experiment of the project, from which all related information, such as experimental protocols or spectral data, is accessible. All analytical protocols, including processing protocol, relating to the spectral data can be accessed through the *Spectral data Interface*. An interactive graphical tool can be used to view either the entire spectrum or to zoom in and focus on one part of the spectrum (Figure 3e). Within a project (when available), all identified and possibly quantified compounds are also available through the *Compounds *menu, via a single web page (Figure 3b and above).

### A knowledgebase for plant metabolites

All the data and metadata deposited in projects (when declared public) are shared with the metabolomics community. Thus, MeRy-B can be used as a knowledgebase. Three helpful tools allow the sorting, visualization and export of the data already stored into the database: the search Spectral Data and search Compound under the tab labeled *Data consult*ation and the Query builder under the *Tools *menu.

The "*Search spectral data*" tool can be used to visualize a MeRy-B spectrum in a matrix of interest (e.g. fruit, seed, leaf, epicarp) from a species of interest or a related species. A multicriterion search of metadata results in direct display of the corresponding spectra. For example, 190 spectra of tomato (*Lycopersicon esculentum*) pericarp obtained on a 500 MHz Bruker Avance at pH 6 in D_2_O solvent were available for public consultation on March 2011. In addition, users can obtain the peak list for each spectrum, the corresponding identified or unidentified compounds and their concentrations. The graphical view of each spectrum is interactive, making it possible to zoom in and focus on a region of the spectrum, to overlay the spectrum and to observe detected peaks. Figures containing NMR spectra in publications are often very small and not interactive. This tool is of particular interest for "beginners" with no experience with a particular tissue or plant matrix. In addition, there are often few published data dealing with the composition of the plant tissue, organ or biofluid and literature searches are time-consuming. MeRy-B currently compiles data for hundred metabolites in four species and eight tissues or organs, together with the corresponding metadata.

**Figure 4:**
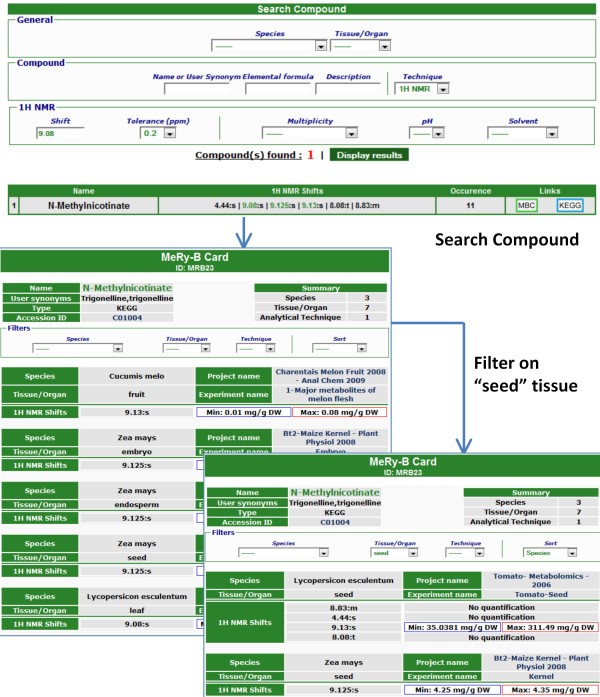
**The MeRy-B card**. The MeRy-B card displays all public data stored in the MeRy-B knowledgebase for a given compound. For each species and tissue in which a given compound is found, this card displays data concerning ^1^H-NMR chemical shifts, multiplicity and quantification. Data may be filtered and sorted by species and/or tissue.

The "*Search compound*" tool enables users to carry out searches of previously detected compounds stored in the MeRy-B knowledgebase. Three types of search may be carried out: (*i*) a compound search (by name, synonym or elemental formula, according to Hill notation), (*ii*) a chemical shift search for ^1^H-NMR data (by chemical shift +/- tolerance, multiplicity, pH, solvent) after the selection of the ^1^H NMR technique and (*iii*) advanced searches corresponding to a combination of both these types of search. For example, a new user observes a singlet at 9.08 ppm in tomato at pH 6. He or she then tries to identify this compound by looking for identified compounds described in the MeRy-B knowledgebase as a singlet close to 9.08 ppm ± 0.2. The search returns one compound: trigonelline, with an external link to the KEGG compound card. The user can then check whether the other three chemical shifts of trigonelline were also detected on his/her NMR spectrum. In addition, another link provides all the information available about each compound in MeRy-B via a "MeRy-B card" (MBC) (Figure 4). Chemical Translation Service (CTS, [53]) and HMDB IDs are also provided when available. For a given compound, the "MeRy-B card" displays the list of experiments in which it was detected and, for each experiment, additional metadata are listed (species, tissue/organ, and project name), together with a summary of the analytical results (e.g. for ^1^H-NMR: chemical shift, multiplicity, minimum and maximum values for quantification). This card also highlights quantitative differences between species, tissues, organs or experiments for a given compound. One or several "MeRy-B cards" are returned for each chemical shift and/or compound search. Comparisons must take into account the possible use of different quantification units. Units are always provided on MeRy-B cards to prevent inappropriate comparisons.

Finally, *Query Builder *is a useful tool for queries and for the export of -omics data. We may need to add to the statistical treatments currently included in MeRy-B, nonlinear unsupervised multivariate methods, such as those based on neural networks, or supervised methods, such as the partial least square (PLS) method, included in tools such as Multi Experiment Viewer http://www.tm4.org/mev/ or MetaboAnalyst [40], or other statistical packages or software. MeRy-B therefore includes a multicriterion search tool for the construction of queries to extract all the corresponding data stored in the database. After initially planning to use BioMART [54], we developed our own query tool with complex filters. Query building is based on the selection of attributes (from project name to compound quantification, multiplicity or chemical shift) collected into logical attribute sets, for selection of the data to extract. Constraints on these attributes can be added, to filter the query results, which are then displayed as an exportable table suitable for analysis with standard statistical analysis tools, such as R software. This query builder has not been developed especially for MeRy-B and is still being developed, to provide a robust and flexible generic tool http://www.cbib.u-bordeaux2.fr/x2dbi/. An example of the use of this module is provided in the Additional file 1.

## Discussion

A number of other databases worldwide are conceptually related to that presented here. However, MeRy-B has several advantages for plant metabolomics and for data management and analysis. MeRy-B is a single tool meeting the needs of the research community in this domain: one or several spectral databases, a knowledgebase for plants with an experimental design description, compound quantification files (when available) and search tools, several tools for spectrum visualization and statistics and one or several metabolite identification tools. These needs were previously met by using a series of databases and applications. Furthermore, MeRy-B was designed to improve the reporting of metabolomics research, based on MIBBI requirements: the MSI. Specialized ontological terms are used where applicable, for experimental design and analytical metadata for NMR, for example. Furthermore, MeRy-B can be used in three main ways: consultation within a project, consultation between projects and consultation of all the data present in the knowledgebase. When compared to human metabolite-oriented HMDB, MeRy-B is metabolomic profiles-oriented and dedicated to plants. When compared to the MetaboAnalyst web tool that handles processed data (peak lists or bucket lists), MeRy-B handles NMR spectra from visualization to statistical analysis using the corresponding metadata.

One key feature of MeRy-B is the *Data consultation *menu, with the *Spectra Overlay *module. Spectra are displayed in color according to the criteria chosen by the user, facilitating the visual inspection and identification of spectral regions varying as a function of the level of a given factor. This ready-to-use tool is much more powerful than the 'dual function' proposed by the manufacturers of NMR software, which is based exclusively on sample code. To our knowledge, this is the only spectrum visualization tool with this overlay feature available.

In publications, NMR metabolomic profiles are generally reduced to one or two representative spectra. These spectra are not interactive and their resolution is often too low for the reader to extract all the information they contain. In this context, MeRy-B is of particular interest for newcomers with no experience with a particular tissue or plant matrix, because it provides access to detailed experimental and analytical protocols, together with the composition of the corresponding plant sample. Such composition data are scarce in publications and their provision by MeRy-B is therefore of great potential utility. As in the HMDB database, the precise tissue or organ distribution of a compound within a plant, together with its quantification, constitute crucial information for MeRy-B users. Indeed, the level of quantification varies as a function of the tissue, organ or species of interest, and users can compare the amounts of a given compound between situations for the identification of potential biomarkers.

In the near future, we plan to make it possible to import and export experiment description data with the emerging ISA-tab format [55], which was developed for the description of investigations, studies and assays for -omics approaches. We will expand the scope of MeRy-B, by extending spectrum management to other analytical techniques, such as GC-MS, LC-MS and ^13^C NMR. The objective is to gather datasets generated by different analytical techniques, making it possible to benefit from their complementarity, as shown by recent publications [56,57]. We also plan to enlarge the MeRy-B knowledgebase by the inclusion of libraries of reference compounds from MeRy-B users or from other available libraries.

## Conclusion

MeRy-B is a web-based application and database for the management and analysis of NMR plant metabolomics profiles, filling the gap in centralized information in this area. This platform manages all the data produced by a metabolomics experiment, from biological source description to compound identification. It also helps the user to analyze and to understand the data, by providing a number of visualization tools, for the visualization of NMR data by spectra overlay or multivariate statistical analyses, for example. By creating integrated visualizations, MeRy-B can provide biological insight. Furthermore, it provides information about metabolite quantification, making it possible to make comparisons between developmental stages, tissues, or environmental conditions. In March 2011, 20 users had a MeRy-B account, and 12 projects, 962 spectra and 100 compounds were available for public consultation in MeRy-B (for an update, see the home page). All these data, cleverly exploited with MeRy-B tools, provide a useful knowledgebase for the sharing of plant NMR profiles and information relating to metabolites. This knowledgebase facilitates the identification of metabolites through comparisons between the spectra obtained for plant extracts and those present in the MeRy-B knowledgebase.

## Availability and requirements

Project name: MeRy-B

Project home page: http://www.cbib.u-bordeaux2.fr/MERYB/home/home.php

Browser requirement: the application is optimized for Firefox. However, it also works satisfactorily with Microsoft Internet Explorer version 7 and Safari.

The user's web browser should support JAVA, to make it possible to benefit fully from MeRy-B.

Users can create an account by submitting a form on the MeRy-B website. The user may populate the database him or herself, or assistance can be provided (see link on the website). MeRy-B is free to all academic users for data submission and their visualization and analysis.

## List of abbreviations

D_2_O: deuterium oxide; DSS: 4,4-dimethyl-4-silapentane-1-sulfonic acid sodium salt; JCAMP-DX: the Joint Committee on Atomic and Molecular Physical data - Data Exchange format; KEGG: Kyoto Encyclopedia of Genes and Genomes. KEGG COMPOUND Database: http://www.genome.jp/kegg/compound/; MS: mass spectrometry; NMR: nuclear magnetic resonance; ppm: parts per million; SOAP: Simple Object Access Protocol; XML: Extensible Markup Language; TSP: (trimethylsilyl)propionic-2,2,3,3-d_4 _acid sodium salt;

## Authors' contributions

ADD and AM initiated the project. HFD, DJ and LG designed the DB. LG and DJ designed the web interface and implemented the DB and associated tools and developed the source code of the web application. CD actively populated the DB, tested the application and tools and provided feedback. HFD, LG, CD prepared the manuscript. DJ and SB participated in the drafting of the manuscript and its figures. CD provided studies for use cases. AM, CD and MN contributed to the critical reading of the manuscript. AM, DR, ADD and MN served as project advisors. All authors have read and approved the final submitted version.

## Supplementary Material

Additional file 1 **One example of use of Query Builder module in MeRy-B**. This workflow tutorial with step-by-step and with screenshots illustrates how to reach the objective of extracting the list of the metabolites identified in the ^1^H-NMR spectra of project T06002: name, chemical shifts, groups and multiplicity. Click here for file
