# Supplementary material for: MeRy-B: a web knowledgebase for the storage, visualization, analysis and annotation of plant NMR metabolomic profiles
Source: BMC Plant Biol. 2011 Jun 13;11:104. doi: 10.1186/1471-2229-11-104 (PMC3141636; doi:10.1186/1471-2229-11-104)
Supplement: Additional file 1 — One example of use of Query Builder module in MeRy-B. This workflow tutorial with step-by-step and with screenshots illustrates how to reach the objective of extracting the list of the metabolites identified in the 1H-NMR spectra of project T06002: name, chemical shifts, groups and multiplicity. [file 1471-2229-11-104-S1.PDF]

Additional File 1: One example of use of Query Builder module in MeRy-B.

<u>Objective</u>: Extracting the list of the metabolites identified in the ^{1}H-NMR spectra of project T06002: name, chemical shifts, groups and multiplicity.

This workflow tutorial with step-by-step and with *screenshots* illustrates how to reach this objective.

Data consultation module

Tools

Query Builder

The Query Builder shows several tabs: *Attributes, Filters, Results Browse*, which should be filled in sequential order. *Attributes* tab allows the user to select amongst all database controlled fields, the fields shown in *Results* tab. ***Add a subset**:* (drop-down list)

Choose *Compounds*

Select the attributes in the *compounds* frame in the order wanted by the user, *i.e.* 1 *user synonym*, 2 *nmr shift description*, 3 *nmr multiplicity* and finally 4 *nmr shift*. The selection order will be the field order in the Result table.

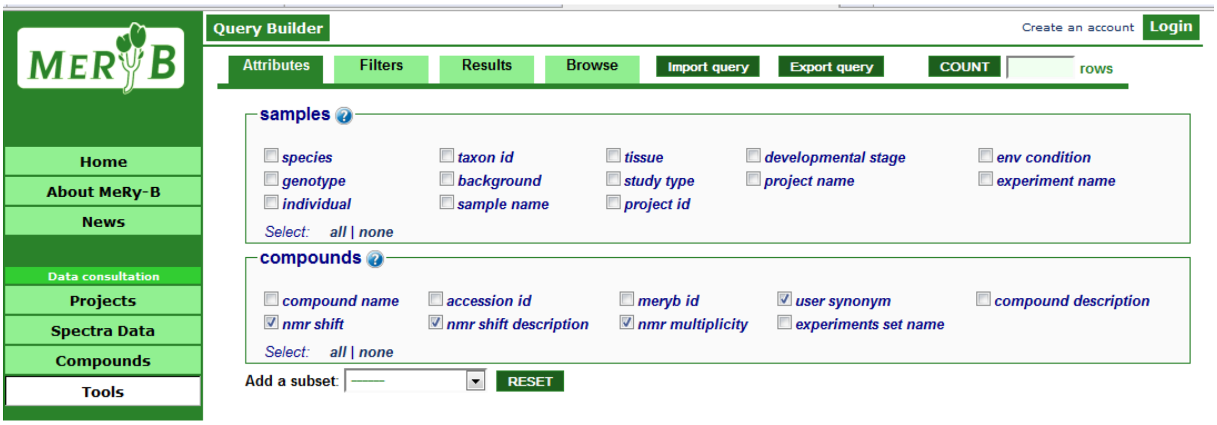

Select the tab *Filters*

This tab allows the user to select a subset of the whole database using filters.

Add a Filter

Select *Project name* in the drop-down list and type T in the last text box.

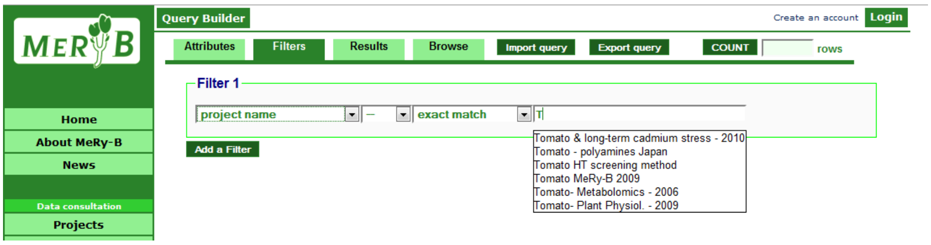

-

Select *Project name* in the drop-down list, *i.e.* Tomato- Metabolomics 2006.

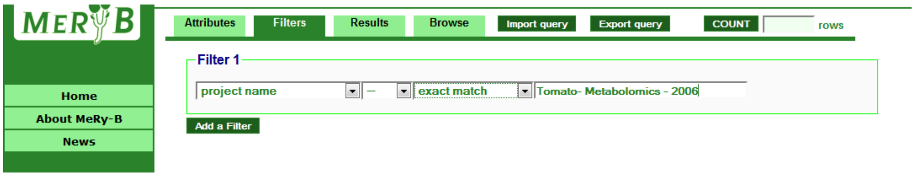

Select the *Results* tab.

This tab allows the user to process the query. The results may be sorted according to a selected field in the *Attributes* tab.

Select *user synonym* in the drop-down list and click on the *Launch query* button.

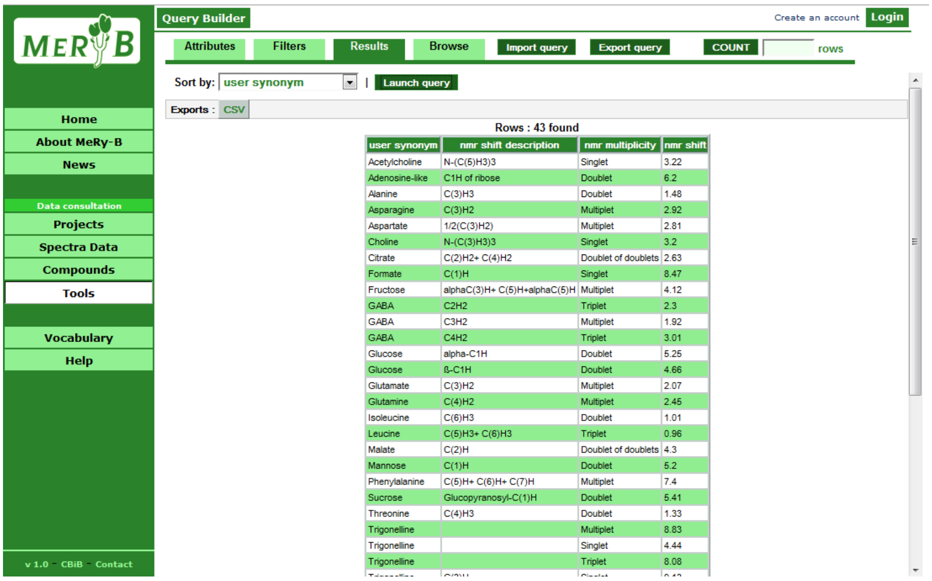

user synonym	nmr shift description	nmr multiplicity	nmr shift
Acetylcholine	N-(C(5)H3)3	Singlet	3.22
Adenosine-like	C1H of ribose	Doublet	6.2
Alanine	C(3)H3	Doublet	1.48
Asparagine	C(3)H2	Multiplet	2.92
Aspartate	1/2(C(3)H2)	Multiplet	2.81
Choline	N-(C(3)H3)3	Singlet	3.2
Citrate	C(2)H2+ C(4)H2	Doublet of doublets	2.63
Formate	C(1)H	Singlet	8.47
Fructose	alphaC(3)H+ C(5)H+alphaC(5)H	Multiplet	4.12
GABA	C2H2	Triplet	2.3
GABA	C3H2	Multiplet	1.92
GABA	C4H2	Triplet	3.01
Glucose	alpha-C1H	Doublet	5.25
Glucose	ß-C1H	Doublet	4.66
Glutamate	C(3)H2	Multiplet	2.07
Glutamine	C(4)H2	Multiplet	2.45
Isoleucine	C(6)H3	Doublet	1.01
Leucine	C(5)H3+ C(6)H3	Triplet	0.96
Malate	C(2)H	Doublet of doublets	4.3
Mannose	C(1)H	Doublet	5.2
Phenylalanine	C(5)H+ C(6)H+ C(7)H	Multiplet	7.4
Sucrose	Glucopyranosyl-C(1)H	Doublet	5.41
Threonine	C(4)H3	Doublet	1.33
Trigonelline		Multiplet	8.83
Trigonelline		Singlet	4.44
Trigonelline		Triplet	8.08

The table can be exported as CSV file by clicking the button *CSV* and opened in a spreadsheet software (e.g. MS Excel), see below. The *Browse* tab shows the user all available fields and field values.

G6 fx

	A	B	C	D	E
1	#43 rows				
2	user_synonym	nmr_shift_description	nmr_multiplicity	nmr_shift	
3	Acetylcholine	N-(C(5)H3)3	Singlet	3.22	
4	Adenosine-like	C1H of ribose	Doublet	6.2	
5	Alanine	C(3)H3	Doublet	1.48	
6	Asparagine	C(3)H2	Multiplet	2.92	
7	Aspartate	1/2(C(3)H2)	Multiplet	2.81	
8	Choline	N-(C(3)H3)3	Singlet	3.2	
9	Citrate	C(2)H2+ C(4)H2	Doublet of doublets	2.63	
10	Formate	C(1)H	Singlet	8.47	
11	Fructose	alphaC(3)H+ C(5)H+alphaC(5)H	Multiplet	4.12	
12	GABA	C2H2	Triplet	2.3	
13	GABA	C3H2	Multiplet	1.92	
14	GABA	C4H2	Triplet	3.01	
15	Glucose	alpha-C1H	Doublet	5.25	
16	Glucose	ß-C1H	Doublet	4.66	
17	Glutamate	C(3)H2	Multiplet	2.07	
18	Glutamine	C(4)H2	Multiplet	2.45	
19	Isoleucine	C(6)H3	Doublet	1.01	
20	Leucine	C(5)H3+ C(6)H3	Triplet	0.96	
21	Malate	C(2)H	Doublet of doublets	4.3	
22	Mannose	C(1)H	Doublet	5.2	
23	Phenylalanine	C(5)H+ C(6)H+ C(7)H	Multiplet	7.4	
24	Sucrose	Glucopyranosyl-C(1)H	Doublet	5.41	
25	Threonine	C(4)H3	Doublet	1.33	
26	Trigonelline		Multiplet	8.83	
27	Trigonelline		Singlet	4.44	
28	Trigonelline		Triplet	8.08	
29	Trigonelline	C(3)H	Singlet	9.13	

Such table is often compulsory in metabolomics publications or reports and the Query Builder tool provides an easy and rapid way to generate it.
